# The Effect of Physical, Biochemical, and Electrical Culture Conditions on Articular Chondrocytes Used for Cartilage Tissue Engineering: A Narrative Review

**DOI:** 10.3390/ijms27156661

**Published:** 2026-07-26

**Authors:** Matthew L. Turner, Ahmad S. Fattouh, Iain S. Whitaker

**Affiliations:** 1 Reconstructive Surgery and Regenerative Medicine Research Centre, Swansea University, Swansea SA2 8PP, UK; 2 Medical School, Swansea University, Swansea SA2 8PP, UK; 3 Welsh Centre for Burns & Plastic Surgery, Morriston Hospital, Swansea SA6 6NL, UK

**Keywords:** chondrocytes, articular cartilage, chondrogenesis, mechano-transduction, extracellular matrix, hypoxia, 3D culture, tissue engineering

## Abstract

Osteoarthritis is a leading cause of disability, most commonly affecting the articular cartilage of the knee in older individuals. Younger people are also at risk of developing post-traumatic osteoarthritis, including athletes and those with a history of joint injury. Focal cartilage defects can be treated through clinical interventions such as autologous chondrocyte implantation, where cells from the patient are isolated, cultured, and subsequently reimplanted into the defect site. Despite recent clinical advances, there is a lack of standardisation in the preparation of chondrocytes for cartilage tissue engineering therapies, with considerable variation in culture conditions reported in the literature. A major challenge in cell-based cartilage repair is the dedifferentiation of chondrocytes during monolayer expansion, which leads to a change in phenotype. Native articular chondrocytes exhibit a rounded morphology and express collagen type II and aggrecan, whilst dedifferentiated cells adopt a fibroblastic phenotype, characterised by an elongated morphology and increased expression of collagen type I. This review summarises the key physical, biochemical, and mechanical factors that mitigate chondrocyte dedifferentiation to support the maintenance of a chondrogenic phenotype and preserve the capacity of cells to redifferentiate. A mechanistically informed approach to chondrocyte culture will enhance cartilage tissue engineering therapies.

## 1. Introduction

Osteoarthritis (OA) is a leading cause of morbidity, affecting over half a billion people worldwide [1]. Historically, OA has been considered a mechanical disease, but its underlying aetiology is heterogeneous and is influenced by inflammatory, metabolic, and post-traumatic processes, which interact with lifestyle and socioeconomic factors [2].

Cartilage has limited regenerative capacity, and surgical intervention is often the only means of alleviating morbidity associated with trauma or disease [3]. Total joint arthroplasty, where the hip or knee is resurfaced with artificial coatings of metal, plastic, or ceramic, is commonly employed when all other treatment options have failed [4,5]. Typically, unsatisfactory remediation is common with increasing age [6,7].

Cartilage defects can also occur in the younger population, and the significant amount of cartilage within the joint can be amenable to alternative surgical interventions [8]. Furthermore, young patients are at significantly increased risk of prosthesis failure and arthroplasty revision and re-revision, compared with older patients [9,10].

For patients with focal cartilage defects, autologous chondrocyte implantation (ACI) offers an alternative surgical procedure for early-stage joint damage [11]. Indeed, the isolation and culture of chondrocytes for ACI, and the development of tissue engineering models, is routine. Yet, culture methods and expansion strategies within the regenerative medicine community can deviate significantly. This review is targeted towards cell and molecular biologists, biomaterials scientists, and researchers with an interest in cartilage repair. We will consider the various culture methods used to expand chondrocytes for reconstructive surgery and tissue engineering, with a focus on physicochemical parameters that circumvent chondrocyte dedifferentiation and promote chondrogenesis, including culture media formulation, growth factor (GF) supplementation, mechano-transduction, oxygen tension, electrostimulation (ES), and three-dimensional (3D) culture.

## 2. Autologous Chondrocyte Implantation

ACI is a two-step procedure used to repair chondral defects within articular joints using the patient’s own cells. First, an osteochondral biopsy is used to extract a small fragment of minor load-bearing cartilage from the patient’s distal femur. The chondrocytes are then isolated and expanded in culture. Second, chondrocytes are injected directly into the cartilage defect, or seeded onto a scaffold material, which is then transplanted into the defect site [12].

**Figure 1:**
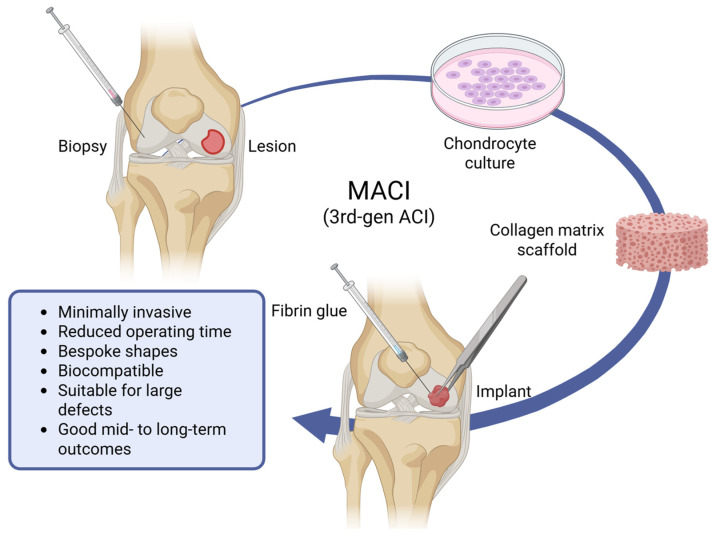
Chondral defect repair by matrix-induced autologous chondrocyte implantation (MACI) (MACI) and its advantages. A biopsy of minimally load-bearing healthy cartilage is extracted, and chondrocytes are isolated for in vitro expansion. Once chondrocytes have substantially proliferated, the cells are seeded onto a collagen matrix scaffold, which is implanted into the defect site. The scaffold is held in place with fibrin glue, and the arthroscopic portal is closed. Created in BioRender. Turner, M. (2026) https://BioRender.com/g5h9qix (accessed on 17 July 2026).

Today, third-generation matrix-induced autologous chondrocyte implantation (MACI) has superseded previous generations and uses a collagen matrix onto which chondrocytes are seeded (0.5–1 × 10^6^ cells/cm^2^). The scaffold is fixed in place with a fibrin glue, or left without fixation, eliminating the need for additional tissue harvest and creating a suture-free solution with shorter surgical times and reduced haemostasis [13]. Each generation of ACI requires the manipulation of cells in vitro, typically through expansion as a monolayer culture, before transferal to a 3D matrix or scaffold (Figure 1).

Fourth-generation ACI introduces a one-step procedure without the need for cell culture. Various implantation strategies have been adopted for this method, such as direct isolation of chondrocytes mixed with allogeneic or autologous mesenchymal stem cells (MSCs) [14]. Others have used chondrons (one or more chondrocytes and the surrounding pericellular matrix) [15] or morselised cartilage, which is subsequently implanted into the debrided defect and fixed in place with fibrin glue [16]. However, the clinical evidence for the efficacy of fourth-generation ACI compared to MACI is limited.

The use of MACI has markedly increased since FDA and EMA approval and is likely the most adopted ACI technique [17]. Tissue-engineered hydrogels are also gaining significant attention in regenerative medicine and have proven effective for repairing spinal cord injury [18]. Although hydrogel therapies such as ChondroFiller^®^ have been approved for the European market, they do not incorporate autologous cells and are typically associated with microfracture augmentation rather than ACI. Furthermore, no hydrogel therapy has received FDA approval.

Although ACI is typically regarded as an intervention for the repair of focal cartilage defects rather than a routine option for advanced OA repair, there is evidence of good short- to mid-term clinical outcomes for patients with early OA, particularly in the young, which delays the need for total joint replacement [11,19]. In fact, younger and physically active individuals can experience soft-tissue injury of the joint, which can lead to post-traumatic OA (PTOA) [20]. Remarkably, up to half of knee joint trauma patients develop PTOA. Furthermore, patients with a prior history of knee trauma are up to six times more likely to develop PTOA and are typically diagnosed with OA 10 years earlier than those without a history of knee injury [21]. Therefore, effective PTOA repair strategies are imperative for ameliorating the development of clinical disease and limiting its progression.

These advancements will be based on the underlying knowledge of native chondrocyte physiology and metabolic adaptations in vitro, particularly when maximising the expansion capability and retention of a clinically relevant phenotype for tissue engineering therapies, such as ACI [22].

## 3. Chondrocyte Metabolism

Cartilage tissue is entirely avascular and aneural, with chondrocytes occupying just 1–10% of the total tissue volume, making cartilage one of the lowest cell density tissues in the body [23]. Chondrocytes are the only mature cell type found in cartilage and exist within a cavity surrounded by extracellular matrix (ECM), known as a lacuna [24]. As the lacuna develops, chondrocytes are enveloped with glycosaminoglycans (GAGs) and collagen fibres, providing cartilage with structural integrity and load-bearing capacity. Adult articular cartilage is composed of more than two-thirds collagen by dry weight [25,26], which imparts tensile strength, whilst sulphated proteoglycans (PGs) attract water molecules and provide compressive strength. Both networks interact synergistically, giving cartilage its unique biomechanical properties [27,28].

**Figure 2:**
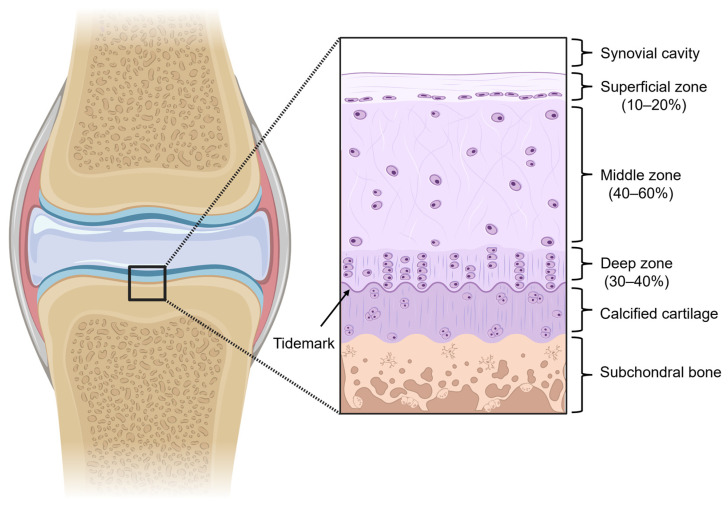
The morphology of articular cartilage. The superficial zone is the thinnest layer and is formed of a dense layer of collagen fibres orientated parallel with the articular surface, along with chondrocytes, which have a flattened morphology. The middle zone, also known as the intermediate/transitional zone, is the thickest layer of cartilage and encompasses 40–60% of the total cartilage volume. Collagen fibres in this zone are less organised and are typically oblique relative to the surface, whilst chondrocytes are more rounded or randomly shaped. In the deep zone, collagen fibres are arranged perpendicular to the surface, and chondrocytes are arranged in stacked columns. The tidemark is a metabolically active and undulating boundary between calcified and uncalcified (articular) cartilage and represents a mineralisation front. Finally, subchondral bone forms the foundation onto which all other layers are directly or indirectly attached. Created in BioRender. Turner, M. (2026) https://BioRender.com/o2r31ou (accessed on 17 July 2026).

Chondrocytes are metabolically active cells that support cartilage homeostasis by regulating the synthesis and turnover of ECM [29]. Nutrients and oxygen are supplied primarily by diffusion from surrounding tissues, principally the synovial fluid [30]. However, as chondrocytes are deeply embedded within the dense ECM, they are subjected to decreased oxygen and nutrient availability compared with cells in well-vascularised tissues, particularly those found in the deep zone (Figure 2).

Chondrocytes consume glucose as a primary substrate for energy metabolism, producing ATP via glycolysis [31]. Glucose is also used as a precursor for the synthesis of structural components within cartilage such as the sulphated GAGs chondroitin sulphate (CS) and keratan sulphate (KS) [32]. The steady supply of glucose is critical for chondrocyte metabolism and ECM homeostasis. The glucose concentration of synovial fluid is not dissimilar to that of blood glucose, albeit somewhat lower, with estimates of around 3.5 mM [33]. However, the glucose concentration in the pericellular environment is as low as 1 mM, while plasma glucose concentrations can reach up to 6 mM [34].

Similarly, oxygen availability within cartilage is substantially lower than in surrounding tissues and decreases with increasing proximity to the bone–cartilage interface [31]. The oxygen tension of synovial fluid has been estimated at a partial pressure of oxygen (pO_2_) of ~7%, while arterial blood exceeds 12% pO_2_ [35,36]. Indeed, there is likely an oxygen gradient ranging from 10% at the cartilage surface to <1% in the deeper zones [37]. This gradient depends not only on the proximity of synovial fluid to vasculature, but also on the balance between the rate of oxygen diffusion through the cartilage matrix and oxygen consumption by chondrocytes. Indeed, chondrocytes occupy a hypoxic, nutrient-limited milieu and have developed a unique metabolic profile, deriving energy primarily from anaerobic glycolysis, which enables ATP production independent of oxygen availability [31,38].

### 3.1. The Crabtree Effect

**Figure 3:**
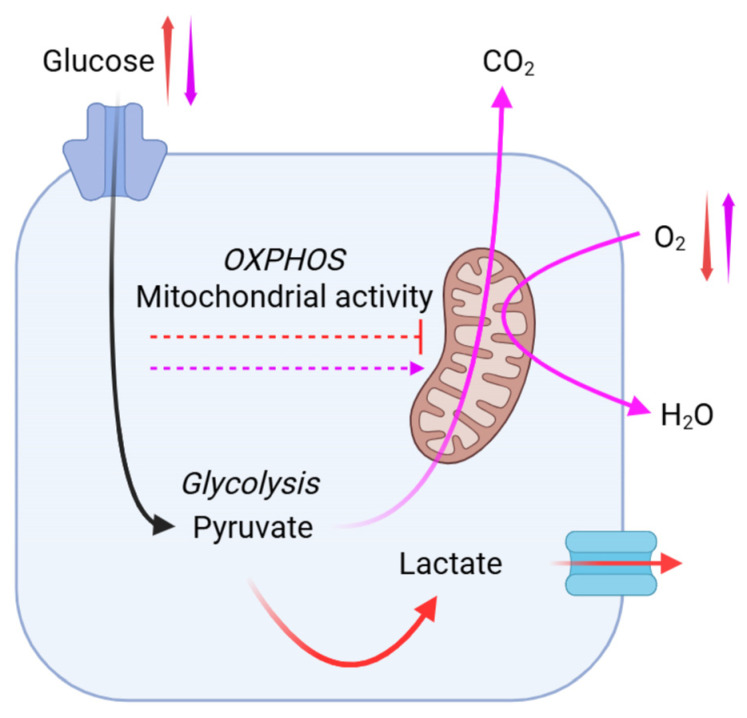
The Crabtree effect in the chondrocyte. An abundance of glucose stimulates glycolysis and the formation of lactate, which is expelled from the cell for oxidation at other tissues. Mitochondrial activity through oxidative phosphorylation (OXPHOS) is reduced, resulting in decreased oxygen consumption (red arrows). Glucose deprivation stimulates OXPHOS and increases oxygen consumption through increased mitochondrial activity, generating carbon dioxide and water as byproducts (purple arrows). Created in BioRender. Turner, M. (2026) https://BioRender.com/8oawjou (accessed on 17 July 2026).

Chondrocytes exhibit a phenomenon known as the Crabtree effect (Figure 3), whereby oxygen consumption increases under conditions of glucose deprivation, and ATP production shifts towards OXPHOS [39]. Conversely, when glucose concentrations exceed a threshold of around 2.7 mM [31,40], oxidative phosphorylation is inhibited, leading to reduced oxygen consumption, as ATP synthesis occurs primarily through glycolysis. This atypical metabolic regulation is likely an adaptation in response to the avascular and hypoxic environment in which chondrocytes reside.

Chondrocytes have a low rate of oxygen consumption, and cartilage tissue consumes as much as 20 to 50 times less oxygen than other tissues [39,40,41]. Around 95% of energy production in chondrocytes is derived from glycolysis [38,42], which is reflected in a low number of mitochondria [43]. Moreover, the low pO_2_ in the cartilage microenvironment stabilises hypoxia-inducible factor (HIF)-1α protein, which is a master regulator of the hypoxic response. HIF-1α stabilisation reduces dependence on oxidative phosphorylation (OXPHOS) and promotes a metabolic shift towards anaerobic glycolysis for energy metabolism [44]. Although glycolysis is a less efficient pathway than OXPHOS for ATP production, it rapidly generates two ATP molecules per molecule of glucose, compared with the ~30 ATP molecules produced more slowly via OXPHOS [45].

### 3.2. Oxidative Stress

In addition to a limited OXPHOS capacity, cartilage has a poor ability to counteract oxidative stress due to its limited antioxidant capacity. During OXPHOS, electrons are transferred along the electron transport chain (ETC), powering molecular pumps that build up an electrochemical potential used for ATP synthesis. Over 98% of the oxygen consumed by mitochondria is used for energy production [46]. However, electrons occasionally ‘leak’ from the ETC and react with molecular oxygen to form reactive oxygen species (ROS), including the primary and secondary species: superoxide (O_2_^•−^) and hydrogen peroxide (H_2_O_2_), respectively. The subsequent breakdown of H_2_O_2_ generates highly reactive hydroxyl free radicals (^•^OH), which are capable of indiscriminately damaging biomolecules, including the glycosidic linkages in cartilage polysaccharides [47].

In healthy tissue, ROS are neutralised by scavenger molecules and antioxidant enzymes to maintain redox homeostasis. By favouring glycolysis over OXPHOS, chondrocytes minimise mitochondrial electron leakage, thereby reducing ROS production and minimising oxidative stress-driven cartilage deterioration. In pathological states, however, mitochondrial dysfunction can disrupt the balance between ROS production and clearance, promoting cartilage degeneration [48]. Metabolic diseases, such as diabetes, can further exacerbate this process by evoking a proinflammatory environment that elevates ROS, which contributes to the progression of OA [49].

### 3.3. Glucose Transporters

Evidence suggests that metabolic disorders, such as diabetes, are intrinsically linked to OA development and progression [50,51]. Chondrocytes express multiple glucose transporters (GLUTs), including GLUT1 and GLUT3 [52], which regulate the uptake of glucose in response to biosynthetic demands and environmental cues such as hypoxia, hormones, and inflammatory cytokines [53,54]. Li et al. developed a mouse model to explore the mechanistic link between impaired glucose metabolism and articular cartilage degeneration. Animals underwent surgical destabilisation of the knee medial meniscus, which resulted in reduced GLUT1 gene and protein expression in the uppermost layer of PG-expressing chondrocytes [55]. This corresponded with OA development, which was accelerated in mice with type I diabetes, providing a causal link between glucose uptake and articular cartilage loss in OA. This suggests that the loss of PG synthesis exacerbates cartilage degeneration. Furthermore, articular cartilage of diabetic mice consumed less glucose and produced less lactate, indicating impaired glycolytic metabolism.

Given that glycolysis is the primary energy pathway in chondrocytes, therapeutic strategies targeting glycolytic metabolism through GLUTs are a realistic prospect for OA treatment [56]. The regulation of glucose metabolism in response to both anabolic and catabolic signals is, in part, mediated by increased GLUT1 expression. One such therapeutic strategy could be the targeting of GLUTs, which are the first rate-limiting step of glycolysis. Lee et al. showed that GLUT1 deficiency results in a decrease in PG, likely due to glucose being the major precursor for PG synthesis [57], the loss of which is an early hallmark of OA. Conversely, sustained GLUT1 expression can be detrimental to joint health through excess production of advanced glycation end products, which degrade cartilage [58]. And so, careful consideration will be required to determine the most appropriate glycolytic candidate targets to achieve the desired therapeutic effect.

### 3.4. Metabolism Summary

Collectively, these observations highlight the unique metabolic phenotype of chondrocytes and emphasise the importance of evaluating the environmental and physiological conditions when culturing cells for tissue engineering applications [59].

## 4. Chondrocyte Dedifferentiation

**Figure 4:**
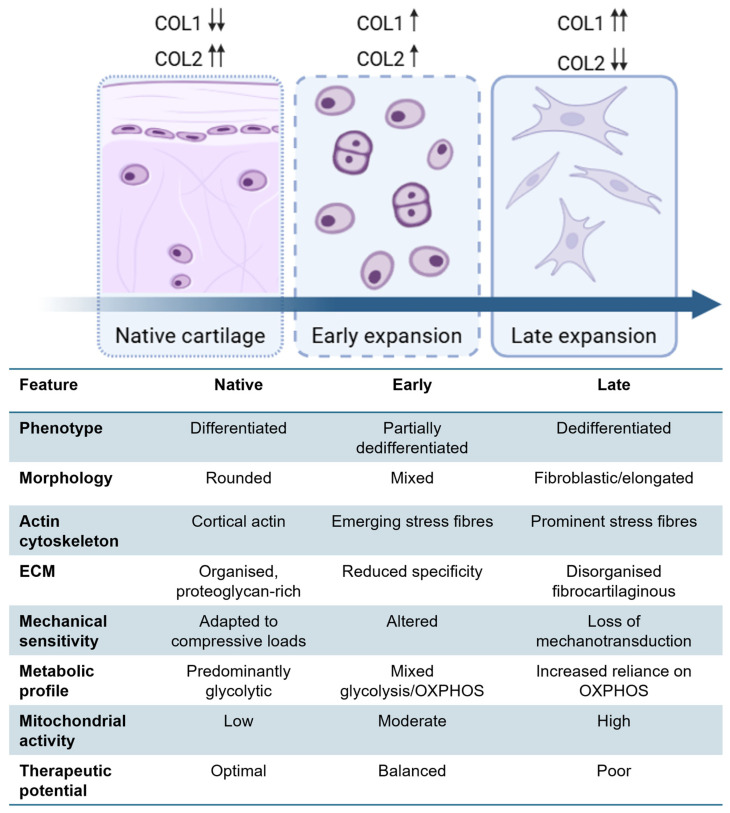
The impact of dedifferentiation on major chondrocyte characteristics including phenotype, morphology, metabolism, and therapeutic potential. Native cartilage has an organised extracellular matrix (ECM) structure with little to no expression of collagen type I (COL1; twin down arrows) and high expression of collagen type II (COL2; twin up arrows). Cells derive energy through glycolysis, and their therapeutic potential is at its greatest. During early expansion, cells transiently shift away from glycolytic metabolism and towards OXPHOS, with decreased expression of COL2 and increased expression of COL1 (single up arrow). In the late expansion stage, chondrocyte morphology becomes fibroblast-like in conjunction with greater expression of fibroblast markers such as COL1. The ECM becomes disorganised, and cells lose their therapeutic potential as they develop an irrelevant phenotype for cartilage repair therapies. Created in BioRender. Turner, M. (2026) https://BioRender.com/fuj2bb8 (accessed on 17 July 2026).

A major biological challenge to overcome during in vitro cell expansion is the ‘dedifferentiation’ of chondrocytes. Articular cartilage chondrocytes classically express collagen type II (COL2) and aggrecan (ACAN) as markers of a chondrogenic phenotype [60]. However, during dedifferentiation, a significant alteration in protein synthesis occurs: the expression of COL2 and ACAN is lost, and cells become fibroblast-like, characterised by increased expression of COL1 and a loss of the transcription factor SOX9, which maintains the chondrocyte phenotype (Figure 4) [61,62,63].

Generally, dedifferentiation takes around five passages of monoculture to occur [61]. There is also reduced deposition of CS, a GAG associated with chondrocytes, but lacking in fibroblastic cell culture [64]. Dedifferentiated cells in 2D monolayer culture also lose their characteristic rounded shape and shift towards a flattened and elongated morphology, closely resembling that of fibroblasts [64,65,66].

### 4.1. Cytoskeletal Reorganisation

Cytoskeletal changes are concomitant with alterations in actin expression and reorganisation. Actin exists as a globular (G) form, which polymerises into filamentous (F)-actin. In primary chondrocytes, actin exists as a cortically arranged filamentous (F) form with a high G:F–actin ratio. The cortex is a thin isotropic network under the plasma membrane, which generates cortical tension when pulled by myosin II motors, generating a rounded morphology [67]. However, monolayer expansion results in decreased expression of the actin-severing protein, adseverin, resulting in elevated actin polymerisation and a decreased G:F–actin ratio [68]. This results in the reorganisation of cortical F-actin into stress fibres, which give dedifferentiated cells a larger elongated spindle shape [69].

Actin cytoskeleton reorganisation actively drives chondrocyte dedifferentiation by reducing the expression of many genes associated with a chondrogenic phenotype [70]. However, it is possible to redifferentiate cells to a differentiated phenotype by restoring the G:F actin ratio through 3D culture, liquid suspension, or in the presence of actin-disrupting agents such as cytochalasin D and latrunculin B [68]. Indeed, overexpression of adseverin restores the G:F–actin equilibrium in expanded chondrocytes and reduces stress fibre formation. Simultaneously, chondrogenic gene expression is increased, whilst COLI is decreased, indicating the capacity of actin-binding proteins to promote redifferentiation [71].

### 4.2. The Physiological Impact of Dedifferentiation

As well as the dedifferentiation of chondrocytes in vitro, there are also changes which occur within the arthritic joint in situ. OA cartilage has at least three chondrocyte phenotypes: activated, hypertrophic, and fibrotic, all of which are distinguished by the collagen markers they express [72]. Activated chondrocytes produce COL2 in the upper and middle zone, whereas hypertrophic cells in the deep zone of fibrillated and eroded OA cartilage express the hypertrophic marker, COLX, which is typically only produced in the foetal growth plate and is absent from healthy joints [73].

COLX is a short-chain non-fibrillar collagen, unlike COL2, which is fibrillar, and has been associated with intrachondral ossification from post-traumatic cartilage lesions [74,75]. Indeed, COL2 was initially regarded solely as a structural component of cartilage. However, COL2 has signalling capability through integrin β1, which suppresses chondrocyte hypertrophy and protects against OA progression [76]. Also present in cartilage are cells with a fibrotic phenotype involved in matrix remodelling that express COL1 and COL3 [77,78]. Recent evidence suggests that destruction of OA cartilage is closely related to chondrocyte dedifferentiation [79].

These morphological, phenotypic, and biochemical changes that occur during dedifferentiation lead to a dysregulation of cartilage homeostasis, resulting in an imbalance between tissue destruction and regeneration [80]. Dedifferentiated chondrocytes produce more catabolic factors, such as matrix metalloproteinases (MMPs) and pro-inflammatory cytokines, which promote ECM degeneration and advance OA progression [72]. In fact, monomeric (non-fibrillar) COL2 induces the expression of MMPs and proinflammatory cytokines, including IL-1β, IL-6, and IL-8. This creates a COL2-dependent feed-forward mechanism whereby the degradation of collagen by MMPs results in the release of COL2 monomers, which, in turn, induce further MMP and cytokine expression, accelerating OA progression in a perpetual cycle [81].

### 4.3. Dedifferentiation Summary

The expansion of chondrocytes in 2D monoculture is a prerequisite for cell-based repair strategies, including ACI [82,83]. The therapeutic success of such procedures is dependent on the preservation of a stable chondrocyte phenotype, or at least the ability of chondrocytes to ‘redifferentiate’ under appropriate conditions [84]. When transplanted into chondral defects, the preservation of a differentiated chondrocyte phenotype capable of forming hyaline cartilage is critical for long-term clinical success [85]. Dedifferentiated chondrocytes produce fibrocartilage, which has inferior mechanical properties compared with hyaline cartilage, leading to poorer repair outcomes [86]. When culturing cells for research or clinical use, the capacity of cells to retain their chondrogenic phenotype and ability to redifferentiate is a major consideration.

## 5. Chondrocyte Culture Media

Chondrocyte culture media formulations are pivotal in directing the fate of isolated primary chondrocytes [87]. Commonly, Dulbecco’s modified Eagle’s medium (DMEM) or DMEM combined with Ham’s nutrient mixture F-12 are used for chondrocyte culture. Not only does medium nourish cells, but it also influences the growth, proliferation, metabolism, and (de)differentiation of chondrocytes [88,89].

The effect of media on cell behaviour and metabolism can be targeted based on the specific composition of the media. Typically, a basal liquid formulation fulfils the cell’s fundamental nutrient requirements, and alterations are made to the principal components, or via supplementation with exogenous metabolites, such as glucose and glutamine, which have a significant impact on metabolic activity [40,89].

### 5.1. Glucose

One of the most ubiquitous chondrocyte culture media is DMEM, which is typically available as a ‘low glucose’ (5 mM) or ‘high glucose’ (25 mM) formulation. The upper estimate for the physiological concentration of glucose in articular cartilage is around 5 mM, and exposure of chondrocytes to supraphysiological concentrations of glucose in vitro influences their metabolic profile and phenotype [40]. As reported in Section 3.1, a glucose concentration threshold of approximately 2.7 mM results in an altered oxygen consumption rate via the Crabtree effect, which has implications for the capacity of cells to form cartilage tissue.

Selecting an appropriate glucose concentration is critical for obtaining the desired effect in culture. Heywood et al. investigated the effect of glucose concentrations on chondrocyte expansion in vitro. Cells cultured in media supplemented with 1 mM glucose exhibited greater expression of chondrogenic markers, including SOX9 and COL2 alpha 1 (COL2A1) chain, whilst having reduced expression of the catabolic enzyme MMP13, compared with cells grown in 10 mM glucose containing media [90]. Similarly, chondrocytes grown as pellets in 1 mM glucose produced greater amounts of ECM compared with pellets grown in 10 mM glucose. There was also a >30% increased population doubling limit, suggesting that lower-glucose conditions preserve chondrocyte differentiation and enhance the ability of cells to form cartilaginous ECM in vitro. Intriguingly, increased ROS were observed following expansion in 10 mM glucose despite a reduced dependence on OXPHOS for ATP synthesis.

These are clinically relevant observations, as a limitation of population doubling is often associated with cumulative cell stress, which compromises the ability of chondrocytes to maintain and repair articular cartilage [91]. Similarly, ROS generation is affected by glucose concentrations and highlights the importance of cell expansion in favourable glucose conditions to minimise oxidative stress, which leads to chondrocyte senescence [91].

Others have used differential glucose concentrations to enrich a subpopulation of chondrocyte-derived progenitor cells (CPDCs), which possess functional cartilage repair, both in vitro and in vivo [92]. Jiang et al. claim to have captured the emergence of CPDCs derived directly from adult human chondrocytes using a low-density, low-glucose 2D culture condition, whereby chondrocytes gradually adopt a stem cell-like phenotype with increased expression of genes associated with MSC maintenance and multipotency. When these cells were seeded into a collagen type I/III scaffold and surgically implanted into the knee joints of young patients with large cartilage defects, clinical evaluation indicated a significant improvement for patient quality of life compared with the preoperative state [92]. Although the source of CPDCs in this study is queried by the authors—whether already present in cartilage tissue or existing as plastic mature chondrocytes, which revert to a stem-like state—it highlights the impact of glucose concentrations on the chondrocyte phenotype and the subsequent implications for therapeutic applications.

### 5.2. Glutamine

Glutamine metabolism plays an important physiological role in chondrocytes, supporting chondrogenic gene expression, proliferation, and matrix synthesis [89]. Additionally, the antioxidant glutamine derivative, glutathione, protects cells against harmful ROS. Data suggests that SOX9 regulates such metabolic pathways and stimulates glutamine metabolism in chondrocytes by binding to the enhancer region of genes involved in glutamine metabolism [89]. Epigenetic modifications of chondrogenic genes, including histone acetylation of ACAN and COL2, render chromatin into an open state, which allows SOX9 promoter binding and chondrogenic gene expression [93].

In vivo, glutamine has been implicated in joint inflammation, which promotes the degradation of cartilage. The inflammatory environment of the OA joint induces metabolic reprogramming, with increased reliance on glycolysis and substitution of glucose with glutamine as a TCA cycle substrate. In fact, culturing chondrocytes in the absence of glutamine decreases cell viability, and the inhibition of glutaminase, which converts glutamine to glutamate for entry into the TCA cycle, mirrors the effects of glutamine deprivation [94].

## 6. Serum Supplementation

Culture media is typically supplemented with serum, which enriches the basal media with a broad spectrum of macromolecules, carrier proteins and trace elements for propagating cells. Serum is rich in lipids, GFs, hormones, and many other metabolites, including glucose, that support cell metabolism, proliferation and phenotype maintenance [95]. However, there is evidence that some sera at high concentrations can promote the dedifferentiation of chondrocytes in 2D culture.

### 6.1. Foetal Bovine Serum

Culture medium is commonly supplemented with up to 20% (*v*/*v*) foetal bovine serum (FBS). Without exogenous GFs, serum is a prerequisite for the expansion for chondrocytes in monolayer culture. Yin et al. recently demonstrated that FBS promotes the proliferation of chondrocytes, increases chondrogenic gene expression, and results in a higher ratio of ACAN- and COL2-positive cells [96]. However, other studies report a more detrimental effect on the chondrocyte phenotype.

Karim et al. seeded cells in soft agarose gels (0.2% *w*/*v*) and cultured them in 1–10% FBS in DMEM. After 7 days of culture, higher concentrations of FBS resulted in an increased proportion of cells with an abnormal morphology, defined as those cells with non-spheroidal shapes and cytoplasmic processes emanating from the cell body, which are commonly associated with fibroblast-like cells [97,98]. Not only was the number of cells with processes significantly greater in 10% FBS, but the number of processes per cell increased in a concentration-dependent manner [99]. Similarly, the density and clustering of chondrocytes was greatest in 10% FBS.

FBS exerts its effects in four sequential stages: (1) an increase in chondrocyte volume; (2) development of cytoplasmic processes; (3) increased cell density; and (4) cluster formation. Cell clusters are a characteristic feature of OA and are associated with increased proliferation [100]. These observations suggest that higher concentrations of FBS accelerate chondrocyte dedifferentiation and underline the importance of selecting the appropriate concentration of FBS for maintenance of the chondrogenic phenotype.

The composition of FBS is often ill defined, and there is a high degree of batch-to-batch variability due to its finite supply, as well as biological variation between source animals [101]. To further confound matters, there are many small-molecule factors in FBS, which remain unidentified [102]. Indeed, serum variability is a major contributor to reproducibility issues in cell biology, with 80% of biologists being unable to replicate others’ experiments and 60% unable to replicate their own results [103]. However, FBS is still the most commonly used supplement in cell culture [104] and has profound effects on the chondrocyte phenotype [96].

### 6.2. Autologous Serum

**Figure 5:**
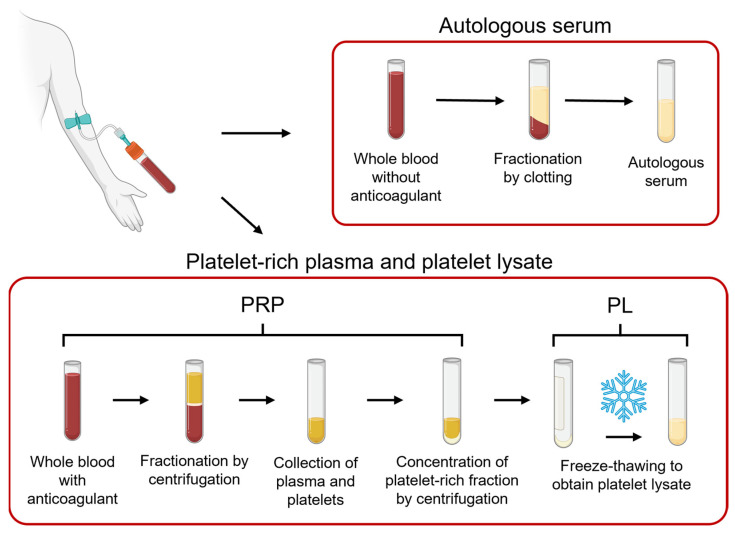
Serum, platelet-rich plasma (PRP), and platelet lysate (PL) preparation from whole blood. Autologous serum is prepared by clotting whole blood and collecting the serum fractionate. PRP is prepared by centrifugation of whole blood and collection of the plasma and platelet fraction. Platelets are concentrated by centrifugation and resuspended in saline solution. For PL, the PRP is subjected to multiple freeze–thaw cycles for platelet lysis. Created in BioRender. Turner, M. (2026) https://BioRender.com/x87zjeo (accessed on 17 July 2026).

One alternative to FBS is human autologous serum (hAS), which is derived from the patient’s own blood (Figure 5). The mitogenic effects of hAS are similar to those of FBS, with increased ACAN and COL2 expression [96]. Whilst FBS is beneficial for the short-term proliferation of chondrocytes, hAS supplementation is advantageous for long-term culture. hAS promotes homeostasis and cell survival through the processing of damaged organelles and suppression of apoptosis, respectively [105,106], whilst autophagy maintains a chondrogenic phenotype in hAS-cultured chondrocytes.

### 6.3. Conditioned Serum

Serum can also be “conditioned” by subjecting hAS to a particular incubation condition to stimulate a biological response. For instance, Jiang et al. showed that when blood from healthy donors was subjected to 4 days of hypoxia, it accumulated a significantly greater quantity of GFs, such as transforming growth factor (TGF)-β1, insulin-like growth factor (IGF)-1, and fibroblast growth factor (FGF)-2, when compared with normal serum from donors [107]. As a result, the number of chondrocytes significantly increased when cultured in 10% hypoxic preconditioned serum (HPS) compared with 10% FBS. Notably, HPS-cultured chondrocytes displayed a significant chondrogenic redifferentiation potential, which was up to 53 times higher than other culture conditions. This was marked by increased immunostaining for COL2, along with increased expression of SOX9 and lower expression of the dedifferentiation marker COL1A1. This demonstrates the differential impact of serum conditioning on chondrocyte expansion and phenotype maintenance.

### 6.4. Platelet-Rich Plasma and Platelet Lysate

Similar products to hAS include platelet-rich plasma (PRP; Figure 5), which consists of a plasma fraction with a concentrated platelet count, again, collected from the patient’s own blood. Despite being anucleate blood cells, platelets are a major source of GFs, with pivotal roles in haemostasis, tissue repair, and immune modulation [108]. This makes PRP an attractive alternative to hAS. Akeda et al. showed that PRP increased chondrocyte proliferation and increased the rate of PG synthesis compared with FBS and platelet-poor plasma [109]. De Angelis et al. demonstrated that the gene expression of COL2A1 was significantly higher in 5% human platelet lysate (PL)-supplemented cultures, whereas COL1A1 expression was significantly lower in 5% PL compared with 10% FBS [110]. Similarly, expression of ACAN and the large CS PG, versican (VCAN), was upregulated in PL-supplemented media.

### 6.5. Serum Supplementation Summary

There are several disadvantages associated with autologous serum products, including limited serum volume; onerous material processing under good manufacturing practices; and a lack of standardisation, with undefined serum compositions due to patient-to-patient biological variability. However, the benefit–cost ratio is favourable when compared with FBS, which is highly variable and introduces additional risks associated with xenogeneic disease.

PL, which is derived by chemical or mechanical lysis of PRP, is a cell-free product, which can be characterised and standardised before use. In fact, lysate can be pooled from multiple donors and stored frozen, offering greater flexibility for its clinical utility [111]. These data indicate that PL supplementation maintains a pro-chondrogenic phenotype and reduces the expression of dedifferentiation markers. Such studies highlight the importance of selecting the appropriate serum type for maintaining a dedifferentiated phenotype or promoting redifferentiation from the dedifferentiated state by mimicking the in vivo biomolecular environment.

## 7. Growth Factors

Growth factors are ubiquitous components of serum and key mediators of its biological activity. Anabolic GFs stimulate synthesis of ECM components such as PGs, ACAN, and collagen, as well as having anti-catabolic activity. The potency of GFs allows for a reduction of serum supplementation down from the traditional 10% *v*/*v* to 2% *v*/*v* [112], or the removal of serum from culture media entirely.

Serum-free media can support chondrocyte expansion in vitro and promote a chondrogenic phenotype whilst simultaneously eliminating the risks associated with xenogeneic sera [113]. Additionally, serum-free media eliminates factors that may adversely affect the efficacy of GF treatment. For example, soluble receptors present in serum may sequester GFs, rendering them latent [114]. Serum may also influence the downstream signalling pathways that are shared between GFs and other bioactive molecules [115].

Although there is no standardised formulation for chondrocyte expansion for specific tissue engineering applications, numerous well-characterised GFs commonly used during culture are listed in Table 1 below.

### 7.1. BMPs

Bone morphogenetic proteins (BMPs) are part of the TGF-β superfamily and are involved in cartilage development during embryogenesis. They directly regulate the expression of chondrogenic genes such as the master transcription factor SOX9, which regulates PG and COL2 production [131,132].

Expanded chondrocytes can be redifferentiated with BMP-2. When Salentey et al. dedifferentiated chondrocytes through multiple passages, they found that treatment with 50 ng/mL BMP-2 promoted a chondrogenic phenotype characterised by upregulation of COL2, SOX9, and ACAN [116]. Preclinical studies in rabbits and rats have also verified the therapeutic potential of BMP-7 in protecting against OA development and progression [133,134]. In fact, BMP-7 has been evaluated in clinical trials for improving the symptomatic response of OA [135]. Although adverse effects did not differ between BMP-7 and placebo groups in the trial, the results of the study were somewhat underwhelming, and the outcomes of the phase II trial were not reported, and no follow-up study has been conducted. However, the clinical relevance of BMP therapy for cartilage repair and regeneration is still very much at the forefront of preclinical studies [136,137].

BMP-7 has anti-fibrotic properties and attenuates chondrocyte hypertrophy. Exposure of OA human articular cartilage cells to 1 nM BMP-7 for 24 h reduced the expression of markers associated with a fibrocartilage phenotype, including downregulation of COL1 [138]. Interestingly, in the study by Ripmeester et al., BMP-7 treatment increased the expression of MMP-2, a COL1-degrading enzyme, and its relative activity in culture supernatant was also significantly higher. Indeed, inhibition of MMP-2 activity via the small-molecule inhibitor OA-Hy resulted in increased COL1 protein relative to the untreated control, which could not be rescued by BMP-7. This indicates that COL1 degradation by MMP-2 is regulated by BMP-7 [138], which has implications for the development of fibrocartilage and the potential for clinical modulation.

### 7.2. FGF-2

Prolonged expansion in a dedifferentiated state reduces the potential for chondrocytes to reverse their phenotype, even becoming irreversibly altered [139]. FGF-2 maintains the capacity of chondrocytes to redifferentiate when removed from expansion culture and introduced to a differentiation environment such as 3D culture. Martin et al. expanded chondrocytes in the presence or absence of FGF-2 and subsequently transferred cells onto a 3D scaffold structure. FGF-2-expanded cells had reduced F-actin fibres, and when seeded onto a 3D mesh, produced more GAG and COL2 than cells expanded without FGF-2 [119]. Thus, FGF-2 preserves the chondrogenic potential of cells in part through changes in the architecture of F-actin filaments.

Paradoxically, FGF-2, in combination with insulin, actually promotes rapid dedifferentiation characterised by reduced COL2 synthesis and stimulates proliferation of chondrocytes [140]. However, the effect is reversible and highlights the ability of FGF-2 to maintain the potential of chondrocytes to regenerate cartilaginous tissue when an appropriate redifferentiation cue is present. This presents an opportunity to use fewer donors or cell passages to achieve the cell densities required for ACI therapy without permanently sacrificing chondrogenic potential.

### 7.3. IGF-1

When cartilage was damaged in a rabbit osteochondral defect model, the knee joints that healed most effectively were those that received a biomaterial implant comprised of laminin and MSCs treated with IGF-1 and TGF-β1 [121]. These animals had improved hyaline cartilage formation with increased COL2 and ACAN gene expression, along with greater histological staining for PGs compared with animals that received untreated implants. The chondrogenic potential of articular chondrocytes expanded in monolayer culture is also markedly preserved for up to seven passages when cells are stimulated with 10 ng/mL IGF-1 at each passage. Without IGF-1 supplementation, chondrocytes were not capable of redifferentiation beyond passage 4 [122].

Interestingly, IGF-1 can inhibit cartilage catabolism. Fosang et al. used the proinflammatory cytokine interleukin (IL)-1α to induce the degradation of PG using an articular cartilage explant model [123]. PG degradation and release increased when explants were exposed to IL-1; however, the presence of IGF-1 (100 ng/mL) reduced PG degradation by up to 86% and reduced soluble GAG loss by approximately half, demonstrating the effective antagonistic properties of IGF-1 against IL-1-induced cartilage degeneration.

The autocrine production of IGF-1 contributes to the maintenance of chondrocytes in vitro and in vivo through mechanisms that promote survival by inhibition of cell death pathways [141]. When cells are treated with neutralising antibodies (Ab) for IGF-1 or IGF-1 receptor (IGFR) in vitro, cell death is significantly increased through enhanced caspase-3 activity, which leads to apoptosis [142]. Evidence also indicates that the responsiveness of chondrocytes to IGF-1 decreases with age. For instance, when old and young chondrocytes are treated with the neutralising antibody for IGFR, there is no appreciable difference in the level of cell death between the age groups. However, when an apoptosis-inducing agent such as ceramide, in combination with IGFR Ab, is introduced, older chondrocytes cannot respond to the survival signals mediated by IGF-1 [142].

IGF-1 is expressed by articular chondrocytes in situ [143] and acts as a potent anabolic factor for cartilage repair, inducing the differentiation of MSCs toward a chondrogenic lineage. However, studies using arthritic mouse cartilage demonstrate a lack of response to IGF-1 [144], which may be due to excessive production of IGF-binding proteins (IGFBP). In fact, human OA chondrocytes produce IGFBP, which has a similar affinity for IGF-1 as IGFR and can prevent IGF-1 from exerting its biological effects [145]. As such, the therapeutic delivery of IGF-1 into OA joints through direct introduction may not be an effective means of treatment due to the presence of IGFBP, and the systemic delivery of such a pleiotropic molecule can have serious adverse side effects.

### 7.4. PDGF

Platelet-derived growth factor (PDGF) is a strong stimulator of chondrocyte proliferation and ECM synthesis [146]. It exists as multiple isoforms; here, we will focus on PDGF-BB, which can activate all three receptor configurations.

Interestingly, PDGF attenuates apoptosis via the ERK 1/2 and p38 intracellular signalling pathways by regulating their phosphorylation status in a dose-dependent manner [147]. The level of apoptosis in vivo is reduced in rat knee OA models, even at the lower concentration of PDGF (10 ng/mL), resulting in a cytoprotective effect [147].

The effects of PDGF foster a chondrogenic phenotype through multiple mechanisms, including inhibition of F-actin stress fibre formation. Recently, Wang et al. showed that PDGF stabilises the hypoxic microenvironment and regulates the cytoskeleton through RhoA/ROCK pathway inhibition and HIF-1α-dependent regulation of adseverin. This leads to depolymerisation of F-actin and maintains the dynamic equilibrium of G- to F-actin typically found in differentiated chondrocytes [125]. Additionally, PDGF has been observed to enhance the expression of cartilage-specific markers whilst reducing the expression of COLX [148,149].

Evidence suggests that the use of PDGF, in conjunction with other GFs, may support the chondrogenic phenotype, particularly in the early expansion stage. Montaseri et al. used an IL-1β-induced dedifferentiation model to demonstrate that PGDF and IGF-1 (10 ng/mL) augmented the downregulation of COL2 and ACAN in primary chondrocytes [150]. Co-treatment with both GFs (5 ng/mL each) further increased the protein expression beyond that of the control, indicating that these GFs act synergistically as anabolic mediators for ACI.

### 7.5. TGF-β

The TGF-β family of proteins includes three isoforms that regulate cell metabolism and have a role in the regulation of chondrocyte behaviour. Notably, TGF-β3 stimulates chondrogenesis in vivo and promotes the chondrogenesis of stem cells such as adipose-derived stem cells and MSCs [130,151]. Like FGF-2, TGF-β3 promotes chondrocyte dedifferentiation in vitro in 2D cultures. However, unlike FGF-2, TGF-β3 reduces articular chondrocyte proliferation and has a negative effect on cell-adhesion strength [152].

Interestingly, the effects of TGF-β3 exposure are markedly different when introduced to cells in 3D culture, particularly through transient exposure. Byers et al. subjected chondrocyte-laden agarose gel discs to TGF-β3 treatment in a continuous or transient exposure model [126]. They found that transient exposure to TGF-β3 for the first two weeks of a 56-day culture period significantly increased the mechanical strength of hydrogel discs compared with discs continuously exposed to TGF-β3 or discs with no exposure to TGF-β3. The increase in Young’s modulus and dynamic modulus was also time- and dose-dependent, with concentrations of up to 10 ng/mL TGF-β3 stimulating increased mechanical strength. However, transient supplementation did not increase total collagen production compared with continuous supplementation. However, sulphated GAG content was rapidly induced and correlated well with increased mechanical strength [126]. These data suggest that TGF-β3 acts to “prime the pump” and exert its effects following its dissipation from the media.

### 7.6. Growth Factors in Combination

The biological effects of many GFs are enhanced by their synergy when combined [153]. Iwata et al. sought to develop a serum-free culture media containing a mixture of GFs for chondrocyte proliferation in cartilage tissue engineering. They incorporated FGF-2 (100 ng/mL), EGF (10 pg/mL), PDGF (625 pg/mL), and TGF-β (5 pg/mL) to replicate GFs in human serum and found that there was a 25-fold increase in chondrocyte growth by day 10 in culture [154].

When OA chondrocytes are cultured in 3D pellets, the size of pellets is significantly increased when cells are exposed to a combination of TGF-β3 and BMP-7, compared with individually treated pellets [155]. Like the study reported by Byers et al., TGF-β3 stimulated the synthesis of GAGs, which was further enhanced through combinatorial treatment. Furthermore, gene expression analysis revealed that ECM transcripts such as COL2A1 and ACAN were significantly increased by approximately 10-fold and 5-fold, respectively, compared with individual GF exposure. Markers for chondrocyte dedifferentiation including COL1A1 and MMPs were also reduced most effectively when TGF-β3 was able to act synergistically with BMP-7 [155]. The combination of these GFs highlights the efficacy of combinatorial treatments to stimulate redifferentiation and offers an opportunity for therapeutic use in patients to readjust the balance between catabolism and anabolism in the OA joint.

### 7.7. Growth Factor Summary

GF expression in chondrocytes is often autoregulated in an autocrine or paracrine manner [156], and its biological effects can be inhibitory as well as stimulatory. GF expression can be regulated by the same GF or differentially regulated by each other. For instance, IGF-1 has little effect on its own expression but is augmented when combined with TGF-β1. Similarly, FGF-2 increases its own expression and is further increased by the synergistic action of TGF-β1 acting in concert with FGF-2. In some cases, GFs have an inhibitory effect on each other, such as the inhibition of IGF-1 expression produced by FGF-2 and TGF-β1 [156]. Nevertheless, a fundamental reason for the incorporation of GFs in chondrocyte culture media during in vitro culture is to suppress dedifferentiation, maintain a chondrogenic phenotype, and support the extent of expansion [119]. Choosing the appropriate GFs significantly augments the therapeutic potential of cells for tissue engineering.

## 8. Physical Culture Environment and Mechanical Stimulation

The physical environment in which chondrocytes are cultured influences many aspects of their physiological and biochemical characteristics, including proliferation, dedifferentiation, and chondrogenesis. Commonly, chondrocytes are expanded in 2D and subsequently transferred to a 3D scaffold, as is the case for ACI. However, culture conditions can also be manipulated between static and dynamic systems.

### 8.1. Static and Dynamic Culture

In vitro, cells are typically grown under static conditions in a stable, stationary, 2D monoculture, adhered to a tissue culture surface. Polystyrene is a typical cell culture substrate. However, its rigidity, which is orders of magnitude stiffer than chondrocyte matrix, promotes dedifferentiation [157].

Increased matrix stiffness correlates with a dedifferentiated phenotype. On flat, hard culture surfaces, chondrocytes spread and flatten into an elongated morphology, triggered by changes in actin polymerisation and stress fibre formation, which promotes proliferation, a fibroblastic matrix, and contractile gene expression, all of which lead to the suppression of chondrogenicity [158]. Such monoculture expansion of chondrocytes in vitro also adversely affects the response to mechanical stimuli [159].

Dynamic culture can influence cellular behaviour through manipulation of the culture environment. Natively, chondrocytes are repeatedly deformed through physical loading from everyday activities. Dynamic loading can increase PG synthesis by stimulating biosynthetic activity [160,161]. Methods such as stretching or fluid-induced shear also result in an increase in PG synthesis and a decrease in degradative enzymes such as MMPs [159]. However, Das et al. found that stretching culture expanded chondrocytes significantly; reduced the expression of genes encoding matrix components, such as ACAN and lubricin; and increased the expression of MMPs relative to primary chondrocytes that had not undergone expansion [159]. However, the effects could be tempered by culturing cells in a “redifferentiation medium” (high-glucose DMEM, insulin-transferrin-selenium, TGF-β2, IGF-1, and ascorbic acid), which drives dedifferentiated cells toward a chondrogenic phenotype.

Such dynamic culture aims to overcome some of the common limitations of static culture and improve the physiological representation of the in vivo environment. Indeed, deformation of reimplanted chondrocytes will occur following ACI, and their response to mechanical load must be physiologically relevant and appropriate for the formation of healthy cartilage tissue.

### 8.2. 3D Culture

Chondrocytes cultured in 3D retain morphological and cytological characteristics similar to those of in vivo cartilage cells, such as isolation in ‘lacunae’ [162]. They also secrete ECM and deposit GAGs into the surrounding matrix, unlike 2D controls, which dedifferentiate into flattened fibroblastic cells.

Fibroblasts are directly involved in wound healing and condense collagen gels by exerting contractile forces on collagen fibrils in the ECM, similar to the mechanism involved in wound closure [163]. Ohsawa et al. found that chondrocytes embedded in collagen gels immediately following dissociation from native cartilage tissue did not cause contraction of the collagen matrix, indicating a chondrogenic rather than fibroblastic phenotype. However, contraction was observed in gels containing cultured chondrocytes, highlighting the similarities of the fibroblast phenotype of dedifferentiated chondrocytes with fibroblasts [164]. Since then, the development of 3D culture systems has gone hand in hand with chondrocyte culture, and numerous 3D culture formats have been created and are listed in Table 2 below.

### 8.3. Bioreactors

**Figure 6:**
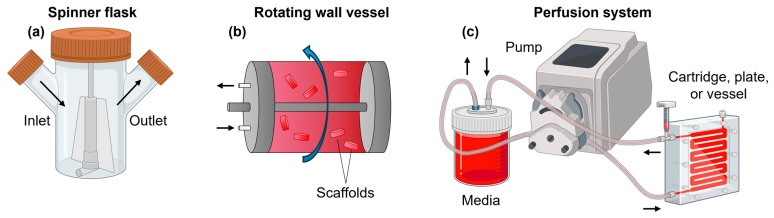
Flow-based cartilage tissue engineering bioreactors. (**a**) Spinner flask, (**b**) rotating wall vessel, and (**c**) perfusion system with laminar flow. Created in BioRender. Turner, M. (2026) https://BioRender.com/668u7xp (accessed on 17 July 2026).

OA tissue engineering strategies commonly employ cell-seeded scaffolds. However, static culture conditions can result in a non-homogenous distribution of cells and ECM within the scaffold and the loss of GAGs to culture medium [177]. Moreover, scaffolds become enveloped in a fibrotic capsule containing multiple layers of elongated cells with little GAG content. This capsule further limits the mass transport of nutrients by diffusion into the core of the scaffold and restricts the extent to which constructs can recapitulate native cartilage [177]. To overcome this, bioreactors are used to mimic the in vivo physicochemical environment with greater fidelity than traditional 2D monoculture systems and exist in many forms (Figure 6). A bioreactor is a device, vessel, or system in which controlled biological or biochemical processes are used to enhance cell proliferation and ECM production via manipulation of pH, temperature, nutrients, pO_2_, and waste removal [178].

Shear stress caused by fluid flow is an important consideration for all types of bioreactors, including bespoke designs, particularly given the sensitivity of chondrocytes to mechanosensation. Vainieri et al. developed a bespoke multiaxial bioreactor, which applies mechanical compression and shear load using a ceramic hip ball to simulate physiological joint kinematics. Chondrocytes seeded onto fibrin-polyurethane scaffolds had significantly increased PRG4, glycoprotein, and a positive ratio of ACAN/VCAN and COL2/COL1. Interestingly, the expression of MMP3 and MMP13 was unchanged [179].

Bioreactors provide a chondrogenic environment in which hydrodynamic forces, combined with the mass transfer of nutrients and metabolites, promote rapid tissue growth and ECM production [180]. Steinwerth et al. used the rotating wall vessel to engineer 3D aggregates without the addition of scaffolds. After 14 days of culture, primary chondrocyte spheroids had reduced COL1A1 and increased chondrogenic gene expression, including COL2A1, SOX9, and PRG4 [181].

Such bioreactor systems can be complex in their design, owing to the complexity of the diarthrodial joint. Nevertheless, they demonstrate the relevance of bioreactors in the development of osteochondral defect models and their necessity in advancing cartilage tissue engineering.

### 8.4. Physical Environment Summary

Mechanical loading is critical for joint homeostasis, with moderate loads contributing to the maintenance of normal tissue function [182,183]. Dynamic stimulation of chondrocytes in monolayer, 3D culture, or as cartilage explants stimulates a coordinated cellular response that brings about changes in gene expression, protein synthesis, matrix composition, and metabolism, which ultimately modulates the biomechanical competence of cartilage tissue [184].

As demonstrated here, there are many ways of introducing mechanical forces to chondrocyte cultures such as shear stress introduced by flow in dynamic culture, or the physical properties of the substrate to which cells are attached. Ultimately, these parameters can be tailored to optimise the chondrogenic potential of cells and improve the therapeutic relevance for ACI.

Physiologically, underloading or overloading the joint both cause cartilage degradation. Indeed, with the onset of OA, there is a general shift of articular chondrocyte activity from a pro-anabolic to a pro-catabolic state [185]. In disease, the balance of tissue repair and restoration is tipped in favour of increased synthesis of MMPs, aggrecanases, and elastase, all of which are cartilage-degrading proteases [186]. Cartilage degradation creates increased stress points in the subchondral bone, which leads to microfractures of the osteochondral junction and underlying bone tissue. This allows synovial fluid to penetrate the subchondral bone and introduce inflammatory mediators that contribute to the pathological changes associated with OA, namely, thickening of the bone (sclerosis).

## 9. Electrostimulation and Bioelectric Properties of Cartilage

Cartilage tissue is ionically charged. During joint movement, fluid flows over fixed ionised macromolecules, provoking strain- and diffusion-generated electric potential. Such electric fields (EFs) contribute to the response of cells to mechanical load and the regulation of cartilage homeostasis. Indeed, degenerative diseases such as OA have disrupted ion flux due to structural deterioration of the tissue, which results in abnormal ion distribution and altered compressive stiffness [187].

### 9.1. Bioelectric Properties of Cartilage

**Figure 7:**
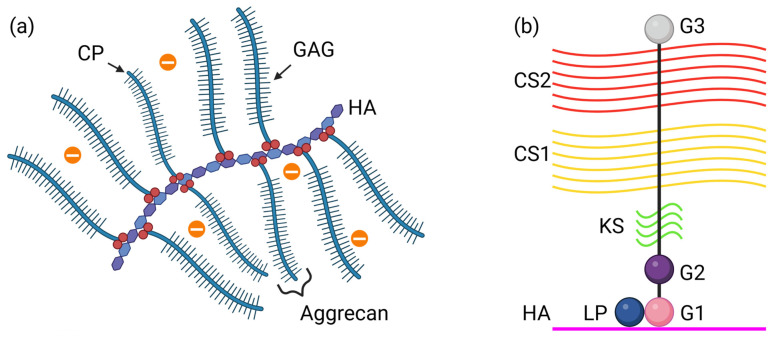
The structure of the proteoglycan, aggrecan. (**a**) Aggrecan (ACAN) is comprised of a central core protein (CP) flanked by glycosaminoglycan (GAG) chains and linked to a central hyaluronic acid (HA) core. Numerous ACAN structures assemble into multi-molecular aggregates that carry a negative charge. (**b**) The ACAN core protein contains three globular domains (G1–G3) and is non-covalently linked to HA by a link protein (LP). Along the protein core are keratan sulphate (KS) and chondroitin sulphate domains (CS1 and CS2). Created in BioRender. Turner, M. (2026) https://BioRender.com/xqrb5xm (accessed on 17 July 2026).

Cartilage is comprised of up to 10% PG, the most abundant of which is ACAN (Figure 7). ACAN forms superstructures by binding with HA. Within ACAN are GAGs that consist of disaccharide subunits that repeat to form unbranched polysaccharide chains of keratan sulphate and chondroitin sulphate, which form KS- and CS-rich domains, respectively. Sulphate (SO_3_^−^), carboxyl (COO^−^), and hydroxyl groups (OH^−^) along these chains carry a negative charge, which facilitates electrostatic interactions. The electrostatic repulsion between GAGs allows cartilage to withstand compression without excessive intratissue swelling [188]. The fixed negative charges in healthy tissue are neutralised by mobile cations in the interstitial fluid. This creates an ionic concentration differential between the fixed solid phase and the fluid phase that results in osmotic pressure, which draws water into the cartilage matrix, providing stability and strength, whilst also contributing to its bioelectric properties [189,190].

### 9.2. Electrostimulation

**Figure 8:**
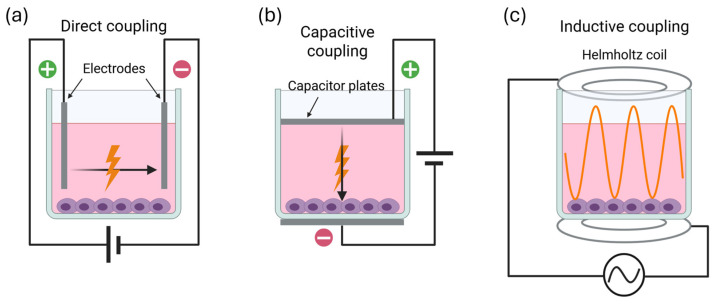
Common methods of electrostimulation for in vitro culture systems. (**a**) Direct coupling, in which parallel electrodes are coupled directly through the culture medium to generate an electric field (EF). (**b**) Capacitive coupling uses conductive plates placed directly above and below the culture system and lie outside of the culture medium. (**c**) Inductive coupling generates oscillating EFs by passing alternating current through conductive (Helmholtz) coils above and below the cells culture vessel. Bold arrows designate EF flow. Created in BioRender. Turner, M. (2026) https://BioRender.com/kazyhq4 (accessed on 17 July 2026).

Electric fields have been applied to chondrocyte cultures in vitro with interesting results. Typically, cells are subject to ES in bespoke culture plates, and electric potential is generated via electrodes or parallel capacitor plates in direct or indirect contact with culture media (Figure 8a,b) [191,192]. Another indirect method of EF generation is inductive coupling, which uses a conductive coil surrounding the cell culture system (Figure 8c).

Intriguingly, EFs stimulate chondrocyte cell proliferation and ECM production through increased synthesis of COL2, GAGs, and ACAN. Hiemer et al. stimulated human knee chondrocytes on bovine collagen-based scaffolds with EFs of 25–30 V/m. Cells were subjected to ES 3 times a day for 45 min over a 7-day period, which resulted in increased expression of COL2 and ACAN mRNA, as well as increased COL2 and GAG protein [192]. Others have reported similar results, including Xu et al., who used capacitively coupled EFs, which induced 4-fold upregulation of COL2 and ACAN, along with 9.6-, 7.1-, or 8.6-fold downregulation of MMP1, 3, and 13, respectively [193].

### 9.3. Electrostimulation Mechanism of Action

**Figure 9:**
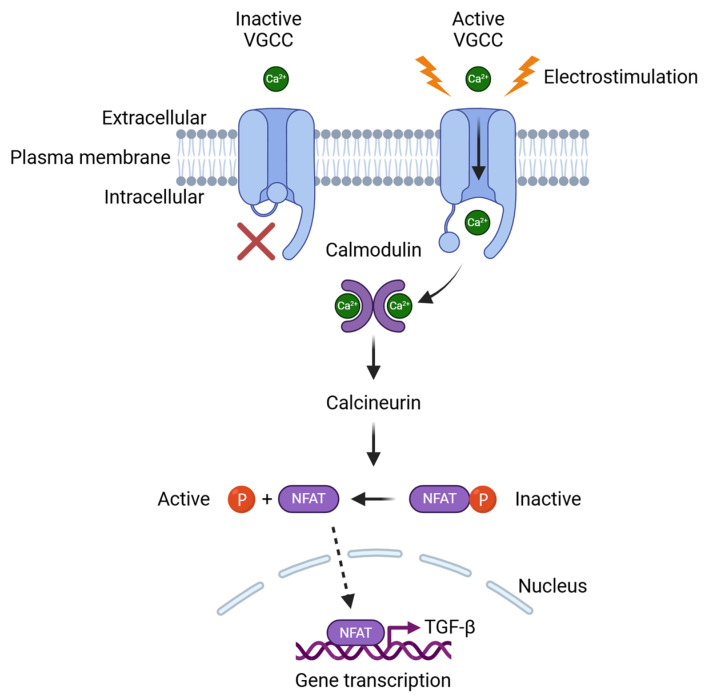
Calcium signal transduction through voltage-gated Ca^2+^ channels (VGCCs) in response to electrostimulation. At resting membrane potential, VGCCs remain in a closed state and prevent the movement of Ca^2+^ ions into the cell. Upon ES, the plasma membrane is depolarised, and VGCCs open, allowing the intracellular influx of Ca^2+^. Calmodulin binds to calcineurin and induces a conformational change that displaces the autoinhibitory domain and activates its catalytic phosphatase site. This results in the dephosphorylation of nuclear factor of activated T cells (NFAT), which translocates to the nucleus (dashed arrow) and enables transcription of target genes such as TGF-β. Created in BioRender. Turner, M. (2026) https://BioRender.com/tic9qjr (accessed on 17 July 2026).

The effects of ES are mediated by the depolarisation of the cell membrane (Figure 9), which activates voltage-gated Ca^2+^ channels (VGCC) that facilitate an intracellular Ca^2+^ influx [193]. Inhibitors of both VGCCs and associated downstream signalling molecules, such as calmodulin and calcineurin, abrogate the physiological response of chondrocytes to ES [193]. On the other hand, the inhibition of intracellular calcium regulation, independent of extracellular calcium influx through VGCCs, does not hinder the effects of ES, further supporting the role of VGCCs in mediating the cellular response to ES. The downstream signalling cascade triggered by Ca^2+^ influx leads to the activation of transcription factors such as nuclear factor of activated T cells (NFAT). The NFAT family is regulated by Ca^2+^ and the Ca^2+^/calmodulin-dependent serine phosphatase calcineurin. When stimulated, NFAT translocates to the nucleus and initiates gene transcription, providing a direct link between Ca^2+^ influx induced by ES, and gene expression [194].

There is evidence to suggest that the transcription of TGF-β is NFAT regulated in some cell types, and that the effects of ES are mediated, in part, by TGF-β [193]. Guerkov et al. used Helmholtz coils to stimulate cells isolated from non-union bone fractures in vitro and found that the pulsed electromagnetic field increased the accumulation of TGF-β in the culture media by 130% after 4 days of treatment [195]. Similarly, chondrogenic in vivo models also demonstrate an increase in TGF-β mRNA and active protein following ES [196]. Indeed, NFATs are key regulators of chondrogenesis and transcribe other GFs. During cartilage development, MSCs condense and differentiate into chondrocytes. Tomita et al. found that, in the presence of elevated intracellular Ca^2+^, calcineurin/NFAT4 activity was a key coordinator of chondrogenesis and stimulated the expression of BMP2 and ACAN protein synthesis [197].

### 9.4. Electrostimulation Summary

Clearly, the application of ES to articular chondrocyte cultures has the potential to inhibit dedifferentiation or to promote the redifferentiation of cells. However, the use of ES during routine culture is not readily employed, owing to the bespoke nature of the electrical culture systems and the expense of purchasing commercially available products. However, such systems can be relatively simple in their design and have a significant impact on the chondrogenic phenotype. This is particularly pertinent when considering the use of chondrocytes for ACI, where the use of EFs can assist in maintaining a positive COL2/COL1 ratio, which promotes hyaline cartilage production for ACI [198,199].

## 10. Chondrocyte Culture Guidance

Based on the evidence presented in this review, we recommend that chondrocyte culture conditions be carefully considered according to the specific experimental objective. It is worth noting that many of the factors described here have been studied in isolation, and their combinatorial effects in culture are less well understood, or unknown. However, there are some central tenets that support the preservation of the chondrocyte phenotype. These include the culture of primary cells in physiologically relevant glucose concentrations, whilst minimising the use of serum or replacing it with chemically defined media to eliminate batch-to-batch variability. Oxygen tension should also be considered, as hypoxic conditions more closely reflect the native cartilage environment that stabilises HIF-1α expression and maintains chondrogenic gene expression. Where serum supplementation is used, it should be batch tested and reported, given its variable composition, including glucose and GF content.

Members of the TGF-β superfamily remain some of the most widely studied GFs for promoting ECM synthesis and preserving chondrogenic gene expression. FGF-2 is also commonly used to enhance proliferation and redifferentiate expanded cells. However, its effects are dependent on dose and timing, and it can cause rapid dedifferentiation in the presence of other growth factors such as IGF-1. During prolonged expansion, the exogenous addition of growth factors is preferable to high concentrations of serum, which can promote chondrocyte dedifferentiation.

Finally, culture configuration is also of relevance. Although 2D culture remains useful for rapid cell expansion, it inevitably results in a progressive loss of phenotype. Consequently, cells should be transferred to 3D culture systems as soon as practical. Pellet cultures and hydrogel-based scaffolds preserve cell morphology, facilitate cell–matrix interactions and promote cartilage-specific ECM deposition. Dynamic culture systems, such as bioreactors, can further enhance these effects by improving nutrient transport and exposing cells to physiologically relevant mechanical stimuli. Dynamic compression, hydrostatic pressure, and fluid-induced shear stress can all positively reinforce the chondrogenic phenotype and limit dedifferentiation.

Although we cannot recommend a specific culture condition for each environmental condition, given the nuances of biological complexity described here, we hope that such considerations will stimulate further research into cartilage repair therapies, which will result in improved therapeutic outcomes for patients.

## 11. Discussion

**Figure 10:**
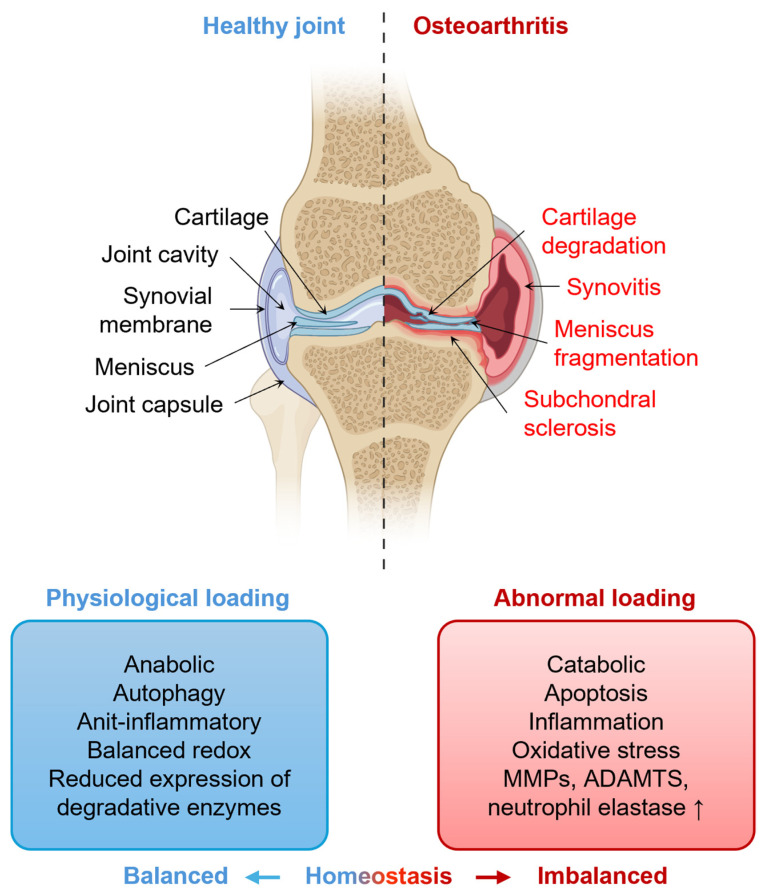
Overview of the impact of abnormal loading in OA. Healthy joints subjected to physiological loading have a balanced rate of anabolism and catabolism, which maintains ECM turnover. Normal loading also has anti-inflammatory properties, which in turn reduces the expression of cartilage-degrading enzymes and maintains a homeostatic environment within the joint. Abnormal loading leads to cartilage degradation and inflammation of the synovial membrane (synovitis). The meniscus is also at risk of fragmentation and accelerates subchondral bone thickness (sclerosis). Over or underloading promotes catabolism and cell death via apoptosis, which contributes to impaired maintenance of cartilage homeostasis. Inflammatory cytokines promote the expression of degradative enzymes, and sustained mitochondrial oxidative stress leads to tissue destruction. Created in BioRender. Turner, M. (2026) https://BioRender.com/rpsp6jd (accessed on 17 July 2026).

Dysregulation of cartilage homeostasis is a fundamental driver of OA. The imbalance between degradation and repair mechanisms leads to the destruction of cartilage tissue, inflammation of the synovial membrane, and sclerosis of subchondral bone, all of which are exacerbated by comorbidities, such as diabetes and obesity (Figure 10). Attempts to restore the anabolic activity of cartilage with small-molecule therapeutics have had little success, with no disease-modifying drugs approved for clinical use. Instead, surgical intervention using total joint replacement, or tissue engineering therapies, such as ACI, are used to repair afflicted joints.

ACI can be an effective method for the repair of medium-to-large focal cartilage defects, particularly in younger individuals with little evidence of advanced OA. The seemingly paradoxical challenge of achieving sufficient cell numbers whilst maintaining a chondrogenic phenotype during expansion can be overcome with strategic cell culture approaches that promote chondrocyte redifferentiation into hyaline cartilage-producing cells.

There is a plethora of culture conditions that influence the chondrocyte phenotype. Many of the studies reported here have been conducted in isolation or with limited experimental variability, as is typical when evaluating the effects of independent variables on dependent variables. However, such factors do not operate in isolation within biological systems. The synergistic (or antagonistic) effects of glucose availability, growth factors, mechanical and electrical stimulation, and dynamic culture conditions on the chondrocyte phenotype have not been fully explored. Consequently, the interaction of these variables and their impact on dedifferentiation remain a considerable unknown, and further research is needed to understand their combined effect.

Preventing chondrocyte dedifferentiation remains a central challenge in advancing cartilage repair strategies such as ACI. Through considered optimisation of the in vitro culture environment, the therapeutic potential of cells to produce hyaline cartilage can be maintained. Collectively, the concepts discussed here highlight how culture conditions and targeted stimulation can be leveraged to preserve the chondrogenic phenotype to enhance the functional quality of tissue-engineered cartilage. Further research and refinement of these approaches will be essential for improved clinical translation of cartilage repair therapies.

## Figures and Tables

**Table 1:** Major GFs implicated in chondrogenesis and cartilage homeostasis, detailing their biological effects and corresponding receptor(s).

	Effect	Receptor	References
BMP-2	Stimulates chondrogenic gene expression and promotes redifferentiation.	Binds to type I and type II BMP receptor	[116,117]
BMP-7	Promotes chondrocyte proliferation and inhibits hypertrophy.	Binds to type I and type II BMP receptor	[118]
FGF-2 (bFGF)	Stimulates chondrocyte proliferation and redifferentiation of expanded cells.	FGF receptor (FGFR)	[119,120]
IGF-1	Regulates cellular proliferation, synthesis, and deposition of matrix proteins including ACAN and COL2.	IGF-1 receptor (IGF-1R)	[121,122,123]
PDGF	Promotes proliferation and inhibition of apoptosis. Inhibits F-actin formation and chondrocyte dedifferentiation.	PDGF receptor (multiple dimer configurations of PDGFR-αα/αβ/ββ)	[124,125]
TGF-β3	Critical role for cartilage formation in vivo. Promotes redifferentiation of chondrocytes in 3D culture. Improves mechanical strength in vitro.	TGF-β receptor 3 (TGFBR3) coreceptor to TGFBR1/2	[126,127,128,129,130]

**Table 2:** The 3D culture systems and materials used for chondrocyte culture.

Type	Method/Materials	Advantages	References
**Scaffold-based**	Chondrocytes are incorporated into a physical material such as a hydrogel or decellularised matrix	Provides physical support for cell growth and deposition of ECM.	[165]
Hydrogel—A network of polymer chains that retains a large volume of water	Alginate Collagen Fibrin Gelatine Hyaluronic acid Poly(ethylene glycol) Polyurethane	Biocompatible, non-toxic, chondrogenic. Tuneable properties such as viscosity, porosity, and mechanical strength.	[166,167,168,169,170]
Nanofibre	Nanocellulose *	Biomimetic structure with properties like collagen.	[166,167,168]
Decellularised matrix	Tissue decellularised ECM Cell-laid matrix	Preserves native tissue architecture. Biocompatible, biodegradable, chondrogenic.	[171]
**Scaffold-free**	Chondrocytes are cultured in an environment that encourages 3D agglomeration in the absence of physical materials	No requirement for foreign, artificial, or natural materials. Physiologically relevant cell interactions. Simplifies regulatory approval and translation.	[172]
Spheroid culture	Hanging drop Static culture Spinner/rotational chamber Concave culture wells	Cells can expand in multiple directions. Cartilage-specific ECM synthesis. Simplified culture system. Enhanced chondrogenesis	[90,173,174,175]
Bioreactor **	Rotating-wall vessels Perfusion systems Spinner flasks	Precise control of mechanical, hydrodynamic, and physical conditions. Enhanced nutrient and oxygen transport. Uniform tissue formation and cell distribution Scalability.	[175,176]

* Nanocellulose is also a hydrogel. ** Bioreactors may be used in combination with scaffold structures.

## Data Availability

No new data were created or analysed in this study. Data sharing is not applicable to this article.

## Abbreviations

The following abbreviations are used in this manuscript:

ACAN	Aggrecan
ACI	Autologous chondrocyte implantation
ATP	Adenosine triphosphate
BMP	Bone morphogenetic protein
Ca^2+^	Calcium
COL	Collagen
CS	Chondroitin sulphate
DMEM	Dulbecco’s modified Eagle’s medium
ECM	Extracellular matrix
EF	Electric field
ES	Electrostimulation
ETC	Electron transport chain
FGF	Fibroblast growth factor
GF	Growth factor
HA	Hyaluronic acid
hAS	Human autologous serum
HIF-1α	Hypoxia-inducible factor 1 alpha
IGF-1	Insulin-like growth factor 1
IL	Interleukin
KS	Keratan sulphate
MACI	Matrix-induced ACI
MMP	Matrix metalloproteinase
MSC	Mesenchymal stem cell
NFAT	Nuclear factor of activated T cells
OA	Osteoarthritis
OXPHOS	Oxidative phosphorylation
PDGF	Platelet-derived growth factor
PG/PRG	Proteoglycan
PL	Platelet lysate
pO_2_	Partial pressure of oxygen
PRP	Platelet-rich plasma
PTOA	Post-traumatic osteoarthritis
ROS	Reactive oxygen species
TGF-β	Transforming growth factor beta
VCAN	Versican
VGCC	Voltage-gated calcium channel
